# Trained Innate Immunity Not Always Amicable

**DOI:** 10.3390/ijms20102565

**Published:** 2019-05-24

**Authors:** Marcin Włodarczyk, Magdalena Druszczyńska, Marek Fol

**Affiliations:** Division of Cellular Immunology, Department of Immunology and Infectious Biology, Faculty of Biology and Environmental Protection, University of Lodz, 90-237 Lodz, Poland; marcin.wlodarczyk@biol.uni.lodz.pl (M.W.); magdalena.druszczynska@biol.uni.lodz.pl (M.D.)

**Keywords:** trained immunity, innate immune memory, epigenetics, atherosclerosis, diabetes, autoinflammatory disorders, neurodegenerative diseases

## Abstract

The concept of “trained innate immunity” is understood as the ability of innate immune cells to remember invading agents and to respond nonspecifically to reinfection with increased strength. Trained immunity is orchestrated by epigenetic modifications leading to changes in gene expression and cell physiology. Although this phenomenon was originally seen mainly as a beneficial effect, since it confers broad immunological protection, enhanced immune response of reprogrammed innate immune cells might result in the development or persistence of chronic metabolic, autoimmune or neuroinfalmmatory disorders. This paper overviews several examples where the induction of trained immunity may be essential in the development of diseases characterized by flawed innate immune response.

## 1. Introduction

For decades, it was believed that the innate immunity arm of the immune system was less sophisticated than the adaptive immunity arm. The primary criterion for such an assessment was the ability of B and T cells, which represent the adaptive immunity, to mount immunological memory [1]. The phenomenon of memory response is at the heart of protective immunization. Interestingly, with the introduction of large-scale vaccination, some effects have been seen that exceeded the intended purpose of immunization. The first observations suggesting nonspecific beneficial effects of vaccination were already related to the smallpox vaccine, when, beyond the desired protective effect against the pathogenic virus, some improvements in the case of e.g., atopic diseases, measles or even syphilis were observed in the vaccine recipients [1,2,3]. Similarly, some nonspecific effects were reported with regard to the BCG (Bacillus Calmette-Guérin) vaccine. As early the 1930s, it was suggested that this vaccine protected not only against tuberculosis but also against other infectious diseases, which could explain the infant survival improvement that exceeded the disease burden of tuberculosis [4]. Studies carried out over the past few decades have shown that certain adaptations connected with innate immune cells (monocyte/macrophages, NK (Natural Killer) cells) are responsible for the nonspecific effects of vaccination [5,6]. In 2011, Netea et al. proposed the term “trained immunity” to describe the ability of innate immune cells to nonspecifically adapt after primary stimulation (e.g., infection or vaccine) and remain at the increased “standby status” for a significant amount of time to be protective against secondary challenge, involving pathogens unrelated to the priming stimuli [7]. It was found that epigenetic reprogramming, mostly connected with histone methylation or acetylation, lies behind the innate immune training [8,9]. Unfortunately, an increasing number of studies provide evidence that epigenetic reprogramming leading to functional and transcriptional changes in the innate immune cells may play a significant role in the maintenance of different disorders.

## 2. Atherosclerosis

It is postulated that trained innate immunity can contribute to the development of some diseases, particularly cardiovascular diseases [10]. In that context, the main attention is focused on atherosclerosis, a pathologically persistent and low-grade inflammation process of the arterial wall and on monocytes/macrophages, the most widely represented immune cells in plaques. The studies conducted by Bekkering et al. (2016) showed that circulating monocytes isolated from patients with symptomatic atherosclerosis, contrary to monocytes of asymptomatic subjects, possessed a pro-inflammatory phenotype characterized by an increased production of interleukin (IL)-6, IL-1β, IL-8, TNF-α (tumor necrosis factor), MCP-1 (monocyte chemoattractant protein-1) after stimulation with LPS (lipopolysaccharide) or Pam3Cys - ligands of Toll-like receptor (TLR)4 and TLR2, respectively [11]. It was found that monocyte reprogramming was expressed by epigenetic modifications related to the downregulation of H3K4me3 on the promoters of pro-inflammatory cytokines, particularly TNF-α, in which case lower H3K27me3 was also noticed. Furthermore, the enhanced cytokine responsiveness was associated with the metabolic shift towards increased glycolysis. These findings have given rise to hypotheses about the engagement of trained innate immunity in the progression of asymptomatic atherosclerotic plaques conversion towards symptomatic disease [11]. The modulations in cell functionality could be the effect of the atherogenic lipid oxidized low-density lipoprotein (oxLDL) induction [12]. Oxidized LDL has been reported to be the inducer of enhanced cytokine production and foam cell formation via epigenetic reprogramming of monocytes. Studies have shown that monocyte pre-exposure to a low concentration of oxLDL followed by stimulation with TLR4/TLR2-agonists results in the amplified expression of numerous proatherogenic proteins, such as IL-6, IL-8, IL-18, TNF-α, MCP-1, as well as metalloproteinase (MMP) 2 and MMP-9. It has been demonstrated that the oxLDL-induced training fails when the monocytes have been pretreated with the histone methyltransferase inhibitor methylthioadenosine. That points to the role of epigenetic modifications in the long-lasting proatherogenetic macrophage phenotype development through oxLDL-induced monocyte training [13].

A better understanding of the epigenetic background of the mechanisms underlying some pathological processes could potentially turn into novel therapeutic strategies, including the treatment of atherosclerosis [14]. In that context, the histone-modifying enzymes seem to be an attractive new target. A few groups of enzymes are the subject of particular interest: histone methyltransferases (HMT) responsible for both lysine and arginine residues methylation, histone demethylases (HDMs) exhibiting opposite activity, and histone deacetylases (HDAC), which counteract acetyltransferases (HAT) activity by removing acetyl-groups from histones [14,15]. It has been demonstrated that through the use of pharmacological inhibitors H3K27me3 demethylases Jmjd3 and Utx, the suppression of LPS-induced production of TNF-α in human monocytes/macrophages is possible. Further, the pre-treatment of monocytes with the methyltransferase inhibitor MTA has been found to disable the oxLDL-derived cell reprogramming. Some expectations are also raised by the use of trichostatin A (TSA), which is a broad-spectrum HDAC inhibitor. Although some results have highlighted its beneficial properties which are characterized by macrophage pro-inflammatory phenotype inhibition, the undesirable effects of the TSA use have also been reported. For instance, the escalation of atherosclerotic lesion formation in LDL-receptor (Ldlr)^–/–^ mouse model of atherosclerosis was noticed after the use of TSA due to the unexpected increase in histone acetylation at the promoter region of cluster of differentiation (CD)36. Thus, the inhibition of particular HDAC enzymes appears to be a better option. It has been described that systemic and bone marrow deletion of HDAC9 is correlated with atherosclerosis limitation in Ldlr^–/–^ mouse model. HDAC9 deletion in macrophages causes the reduction of pro-inflammatory gene expression. Additionally, the enhanced cholesterol efflux and decreased foam cell formation due to the increased expression of cholesterol transporters possessing ATP-binding cassettes for active transport: ABCA1 (cholesterol efflux regulatory protein), ABCG1 (intracellular cholesterol transporting protein), as well as PPARG1 (peroxisome proliferator-activated receptor gamma) have been observed [10,15]. Also, the inhibition of another enzyme from the same group, namely DHAC3, which promotes macrophage polarization toward M1 phenotype, seems to be an interesting solution. In macrophages lacking histone deacetylase 3, the deficiencies in pro-inflammatory activity are observed, which leads to the development of M2-like phenotype of these cells. The silencing of inflammatory responsiveness of monocyte/macrophages could be beneficial in the context of atherosclerosis. It has been demonstrated that it is possible to influence the atherosclerotic plaque formation by HDAC3 manipulation. Although HDAC3 deletion results in the increase in the lesion size, the phenotype of the lesion becomes more stable and is characterized by a collagen-rich fibrous cap, a decreased lipid content and a reduced amount of plaque macrophages [10,15]. Pharmacological, cell-specific intervention to target HDAC3 might be beneficial also in other diseases, including diabetes as it was reviewed by Meier et al. [16].

## 3. Diabetes Mellitus

Diabetes is a complex disease encompassing a wide range of metabolic disorders. Traditionally diabetes mellitus has been divided into type-1 and type-2 diabetes (T1D and T2D, respectively). In the case of the former, one the crucial role is played by the autoimmune response of adaptive immunity (more similar genetically to other autoimmune diseases, particularly to juvenile idiopathic arthritis), while the latter is characterized by insufficient secretion of insulin from pancreatic β-cells, whereby the increase in the production of glucagon by pancreatic α-cells is frequently observed [17,18]. Many factors are indicated to influence the development of diabetes; beyond environment and genetic background, epigenetic modifications have been identified as prominent determinants of the disease outcome. The study with the use of genome-wide DNA methylation quantitative trait locus (mQTL) analysis identified several hundred cytosine-phosphate-guanine (CpG) sites, including known diabetes loci, e.g., ADCY5, KCNJ11, HLA-DQA1, INS, PDX1, GRB10, showing significant association with the insulin secretion and diabetes risk [17]. Functional analyses revealed that the pivotal biological processes such as proliferation and apoptosis in pancreatic β-cells could be under direct influence of some genes: GPX7, GSTT1, SNX19 [17]. In the epigenome-wide association study with the participation of 52 monozygotic twin pairs selected in terms of the discordancy for TD1 occurrence in the immune effector cells such as CD4+ T cells, CD19+ B cells and CD14+CD16- monocytes the remarkable concordance between twins of each pair consistent with a strong shared genetic and non-genetic effect on CpG methylation in strongly inherited DNA promoter regions was shown [17]. Miao et al. demonstrated a significant increase in H3K9me2 in a subset of genes in lymphocytes but not in monocytes isolated from T1D patients compared to the healthy subjects indicating the association between T1D and altered histone methylation of genes playing a crucial role in the processes related to the T1D outcomes [19]. Differences between T1D patients and control individuals were observed also in the acetylation levels of histones H4 and H3K9, and in the level of H3K9me2 in the T1D-related gene CTLA4 [20]. There are strong indications to believe that epigenetic modifications could also be key players in the T2D-related pathways/events as can be seen by the hypermethylation of promoter regions of several genes (INS—encoding insuline, PDX-1—playing an important role in the development of pancreas and in β-cell function, PPARGC1A—participating in the energy homeostasis, GLP1R—regulating insulin secretion) in islets from T2D-donors compared to islets from control subjects [18]. Further, it was shown that hypermethylation and overexpression of HDAC7 (encoding histone deacetylase, HDAC) in islets from T2D-patients can be linked with the impaired mitochondrial function and insulin secretion [18]. Analysis of DNA methylation in samples of liver tissue, visceral and subcutaneous adipose tissues, and peripheral blood from individuals with obesity, with and without T2D conducted by Bajaras-Olmos et al., provided additional evidence that aberrant DNA methylation of genes involved in pathways related to metabolic processes could be a crucial influencer in T2D pathogenesis [21]. In patients with T2D, elevated histone H3 acetylation of TNF-α encoding gene and COX-2 in the blood mononuclear cells, as well as an increase in H3K9me2 near promoter of IL-1A, and PTEN coding regions and Set7-dependent monomethylation of lysine 4 of histone 3 on promoter genes were detected [20].

Cardiovascular disorders are among the most frequently reported complications associated with diabetes mellitus. It is suggested that hyperglycemia could play a role of a long-term activating factor that affects the cellular metabolism of monocytes and macrophages. Epigenetic reprogramming alters the cell functional program to “pro-inflammatory mode”, which has important implications for the occurrence of diabetes complications, including the development of atherosclerosis [22]. It has been shown that monocytes treated with high glucose doses respond with excessive production of IL-1β and IL-6. The secretion of other cytokines, such as interferon (IFN)-γ, IL-17 and IL-22 is also raised, although to a lesser extent. Hyperglycemic conditions could also affect the cytokine production by monocyte-derived macrophages, which after stimulation with LPS or *M. tuberculosis* lysate react with a dose-dependent enhancement in both pro-inflammatory (TNF, IL-6) and anti-inflammatory (IL-10 and interleukin-1 receptor antagonist (IL-1RA)) cytokine release [23]. Additionally, the pro-inflammatory effect of a high glucose environment has been reported in reference to vascular endothelial cells. Studies conducted with the use of bovine and human aortic endothelial cells have shown their epigenetic reprogramming through increased H3K4 monomethylation at the NF-κB (nuclear factor kappa light chain enhancer of activated B cells) promoter, which results in the production of reactive oxygen species (ROS) and upregulation of p65, MCP-1 and VCAM-1 (vascular cell adhesion molecule 1). Those pro-inflammatory features underlie the vascular damage. It is interesting to note that the hyperglycemia-induced pro-inflammatory properties of vascular endothelial cells can persist, even after the cell transfer to the medium with normalized glucose concentration. This phenomenon has been termed “metabolic/glycemic memory” [24]. It has been described that monocytes isolated from both type-1 and type-2 diabetes mellitus patients exhibit functional modifications referring to altered cytokine production and increased binding to endothelial cells. The intensified adhesion to endothelium is most likely responsible for extended migration of monocytes in atherosclerotic plaques. Indeed, the enhanced infiltration of plaques by macrophages in T1D and T2D patients has been demonstrated. It is possible that circulating monocytes in hyperglycemic conditions undergo a training, which “inscribes” their “proatherosclerotic mode” before they infiltrate the atherosclerotic plaque and next this epigenetically programmed phenotype is revealed after monocyte differentiation to macrophages to subsequently encounter with other stimuli, such as oxLDL [15]. The understanding of the epigenetic regulation underlying monocyte-to-macrophages differentiation and trained immunity is a challenge that may deliver new tools to modulate immune response [25].

## 4. Chronic Inflammatory Disorders

Trained immunity has been also shown to participate in the pathophysiology of autoimmune or autoinflammatory diseases. Excessive activation of innate immune mechanisms leading to the enhanced immune response may result in the induction and maintenance of chronic inflammatory disorders such as rheumatoid arthritis (RA), systemic lupus erythematosus (SLE), multiple sclerosis (MS) or sarcoidosis.

### 4.1. Rheumatoid Arthritis

Rheumatoid arthritis (RA) is a chronic, systemic autoimmune disease that is characterized primarily by progressive joint destruction, but in more than 20% of cases it has a profound effect on other organs of the body including the lungs, heart and blood vessels, kidneys or eyes [26,27]. RA disease progression is a complex process that involves interactions between components of both the adaptive and innate immune responses. Cells of the innate immune system, mainly macrophages, are important effectors of tissue-damaging inflammatory lesions, which act through phagocytosis, antigen presentation, and the release of pro-inflammatory cytokines, reactive oxygen intermediates and matrix-degrading enzymes [27,28,29]. The pathophysiology of the disease has not been fully explained; however, it is believed to involve a combination of genetic and environmental factors. Epigenetic mechanisms including posttranslational modifications of histones (acetylation, methylation, phosphorylation, ubiquitination and SUMOylation), DNA methylation, as well as interference of noncoding RNAs (miRNAs), which determine the chromatin state and regulate the accessibility of DNA for transcription factors, have been found to contribute to the pathogenesis of RA by affecting the behavior of several cell types and modifying their gene expression levels [26,30,31,32].

Much evidence suggests that modifications of histones play an important role in the regulation of hyperplasia in the synovial joint [33]. The best studied histone modification is acetylation of lysine residues of histones H3 and H4. The acetylation catalyzed by histone acetyltransferases (HATs) has been found to be associated with enhanced gene transcription, while the deacetylation performed by deacetylases (HDACs) leads to a silencing of affected genes [34]. Most of the available data on the role of histone acetylation in the pathogenesis of RA come from the research using HDAC inhibitors (HDACis). One of them is streptomyces metabolite trichostatin A (TSA), which acts as an inhibitor of class I (HDAC1, HDAC2, HDAC3, HDAC8) and class II (HDAC4, HDAC5, HDAC6, HDAC7, HDAC9, HDAC10) HDACs [26,27]. TSA has been shown to sensitize RASF (RA-derived synovial fibroblasts) to tumour necrosis factor related apoptosis-inducing ligand (TRAIL)-induced apoptosis and promote a cell cycle arrest by inducing the cell cycle regulator p21 [35]. Grabiec et al. demonstrated that two HDACis, TSA and nicotinamide, induced apoptosis also in RA macrophages by specific downregulation of the antiapoptotic protein Bfl-1/A1 and potently blocked IL-6 and TNF-α production [36]. The confirmation of HDACis beneficial effects in arthritis animal models caused these compounds to be recognized as potential therapeutics of RA [37,38,39]. However, it should be remembered that the effects of HDACis are related to histone modifications, because HDACs targets are not only histones but also other proteins including gene transcription factors.

Besides acetylation, methylation or demethylation as well as ubiquitination and SUMOylation of histones belong to epigenetic modifications described in RA [26,29,32]. Depending on the methylated position, the methylation process has been found to be associated with transcriptional activation or silencing [26]. The silencing of genes is associated with trimethylation of histone 3 lysines H3K27m3, H3K9m3 and H4K20m3, whereas H3K36m3, H3K4m3 and H3K79m3 are responsible for the induction of gene transcription [40]. Trenkmann et al. demonstrated that the chronic inflammatory environment of the RA joints induced in RASF the histone methyltransferase enhancer of zeste homologue 2 (EZH2), which added up to three methyl marks to H3K27 of genes designated for silencing, and thus might have caused changes in the epigenetic programme of synovial fibroblasts [41]. Other post-translational processes modulating epigenetic mechanisms in RA are ubiquitination, a process of adding ubiquitin peptides to lysine residues, and SUMOylation, a process that targets proteins harboring a SUMO (small ubiquitin-like modifier) interaction motif [32]. In contrast to histone acetylation and methylation, histone ubiquitination and SUMOylation result in larger covalent modifications of histones. Ubiquitination is associated with transcriptional silencing, initiation and elongation, whereas histone SUMOylation is associated with repressive functions [31]. There is evidence that SUMO is overexpressed in both the synovial tissue and RASF [42]. DeSUMOylation of RASF has been demonstrated to diminish the levels of histone acetylation leading to a lower expression of MMP-1, thus reducing the invasiveness of RASF [43]. Many of epigenome modifiers can directly or indirectly affect the activity of the transcription factor NF-κB, a central regulator of the transcription of many inflammatory genes [44]. The key epigenetic signal for the recruitment of NF-κB to the cytokine genes promoter is the phosphorylation of serine 10 in the tail of histone H3. Glant et al. found that genes encoding Aurora kinases A (AURKA) and B (AURKB) were strongly upregulated in mononuclear cells from both mice and humans with arthritis, which was correlated with elevated levels of phosphorylated H3 [45]. Treatment with the Aurora kinase-specific inhibitor VX-680 attenuated inflammatory reactions and promoted the apoptosis of B cells in arthritic mice [45].

DNA methylation involves the covalent modification of the 5th carbon in the cytosine residue within CpG dinucleotides, which are located close to the transcription start sites of many genes [46]. The methylation of these CpG-rich regions blocks the transcriptional activity of the corresponding genes and results in long-term gene silencing [31,47]. A number of hypo- and hyper-methylated genomic regions have been found in peripheral blood mononuclear cells (PBMC) from RA patients, in RASF, the main cell type invading the cartilage, as well as in RA-derived synovial tissues [40,48,49,50]. Hypomethylation has been shown to be a consequence of an increased metabolism of polyamines, resulting in decreased levels of a methyl donor, S-adenosyl-L-methionine [40]. Most of identified hypomethylated genes have been found to be involved in cell adhesion, leukocyte recruitment, extracellular matrix interactions and inflammation [49]. Nile et al. and Fu et al. showed demethylation in the promoter region of the IL-6 and IL-10 genes, which resulted in the elevation of the cytokines levels during the disease [51,52]. Hypomethylation of the promoter region of the chemokine CXCL12 gene and the retrotransposon LINE1 gene was reported by Karouzakis et al. and Neidhart et al. [53,54]. On the other hand, some DNA regions in RA patients can also be hypermethylated. Hypermethylation of the promoter region of the death receptor 3 gene (DR3), a member of the apoptosis inducing Fas gene family, results in a higher resistance of RASF to apoptosis [55]. RA-derived SF are able to produce proinflammatory cytokines and chemokines as well as matrix metalloproteinases attracting inflammatory cells to the synovium and taking part in the destruction of the cartilage. Lefevre et al. demonstrated that RA-derived SF implanted together with healthy human cartilage into severely immunodeficient mice migrated to the sites of implanted cartilage and destroyed it [56]. The cartilage damage occurred without the development of either cellular or humoral immune responses, suggesting that RASF were capable of maintaining their activated phenotype, characterized by the expression of proto-oncogenes, antiapoptotic molecules and a lack of expression of tumor suppressor genes, in the absence of any further immune stimulation [26,57].

Small non-coding single-stranded RNA molecules (miRNAs) have been recognized as downregulators of gene expression on the post-transcriptional level [27]. Several studies have detected altered expression patterns of miRNAs in RA. Stanczyk et al. showed upregulation of miR-155 and miR-146a in RA synovial fibroblasts and synovial tissues. Monocytes in the peripheral blood of RA patients were also characterized by increased levels of miR-155, whose expression was further enhanced by TNF-α, IL-1β and TLR2, 3 and 4 ligands [58]. Furthermore, the enhanced expression of miR-155 was found to repres the expression of MMP-3 and MMP-1, suggesting its role in the inhibition of the destructive behavior of RASFs [58].

### 4.2. Systemic Lupus Erythematosus

Systemic lupus erythematosus (SLE) is a multisystem autoimmune disease characterized by the development of autoantibody response to nuclear and cytoplasmic antigens. The signs and symptoms of SLE can involve many organs and systems, including the joints, kidneys, lungs, skin, heart and central nervous system [59]. It has been found that epigenetic modifications, in particular DNA methylation and post-translational histone changes, play a critical role in the pathogenesis of SLE. There are a number of studies reporting global DNA demethylation in T cells from active lupus patients, which results in the increase in T cell reactivity [50,60,61]. A possible mechanism explaining DNA hypomethylation is a reduction in the enzymatic activity of DNA methyltransferases (DNMTs) [62]. Deng et al. demonstrated decreased mRNA expression of DNMT1 in SLE-derived T cells, whereas Luo et al. showed a reduced expression of both DNMT1 and DNMT3a mRNAs in active lupus patients [63,64]. DNA hypomethylation referred to many autoimmune-related genes including those encoding CD11a (ITGAL), CD70 (TNFSF7), CD40L (CD40LG) and perforin (PRF1) molecules [65,66,67]. Moreover, genome analysis showed that interferon-related genes such as IFIT1, IFIT3, IFI44L, TRIM22 and BST2 are hypomethylated in CD4+ T cells of SLE patients [68]. Treatment of normal T cells with 5-azacytidine (5-azaC), one of the demethylating drugs, caused global DNA hypomethylation resulting in altered gene transcription and an increase in the expression of CD11a [69,70]. The hypomethylation of IL-4 and IL-6 promoters was found to correlate with IL-4 and IL-6 overexpression, and finally, with the severity of SLE [71].

Modifications of histones have been widely studied in both human and animal models of SLE [48,72,73,74,75]. Monocytes and T lymphocytes of SLE patients demonstrat aberrant histone acetylation and methylation patterns [76]. SLE monocytes show overall H4 hyperacetylation, which results in the upregulation of genes such as IRF1, RFX1 and BLIMP1 (PRDM1) [75]. On the other hand, SLE-derived CD4+ T are characterized by decreased global acetylation of H3 and H4 and decreased global methylation of histone H3K9, which consequently skews gene expression [73,75]. T cells also show increased H3 acetylation at lysine 18, increased H3 dimethylation at lysine 4 (H3K4me2) as well as increased H3 trimethylation at lysine 27 [75,77,78,79]. H3 hyperacetylation and increased H3K4me2 levels in the CD70 gene promoter have been noted to correlate positively with SLE disease activity [79].

SLE patients are characterized by unique microRNA expression profiles involved in the processes of hyperactivation of the type-I IFN pathway, down-regulation of DNA methylation by inhibiting DNMTs and exacerbation of inflammatory responses by promoting the secretion of cytokines and chemokines [76]. In SLE-derived PBMC the expression of miR-189, miR-61, miR-78, miR-21, miR-142-3p, miR-342, miR-299-3p, miR-198, miR-298 shows an increase, while the expression of miR-196a, miR-17-5p, miR-409-3p, miR-141, miR-383, miR-112, miR-184 is reduced [80]. The expression of two miRNAs—miR-21 and miR-148a, which promote CD4+ T cell hypomethylation, has been found to be upregulated in CD4+ T cells from both humans and mice with lupus [81]. Another upregulated miRNA is miR-126, whose overexpression causes demethylation and upregulation of the CD11a and CD70 genes leading to T cell and B cell hyperactivity [82]. On the other hand, miR-125a is significantly downregulated in SLE PBMCs contributing to the elevated secretion of RANTES (CCL5) by T cells [72]. A causal role in the pathogenesis of SLE may also be played by miR-3148, which targets TLR7 mRNA through binding to its untranslated region (3’UTR) resulting in excessive activation of the innate immune system [72]. Recent data have revealed that distinct expression signatures of miRNAs in SLE PBMC are associated with the production of different autoantibodies [83].

### 4.3. Multiple Sclerosis

Multiple sclerosis (MS) is an inflammatory and neurodegenerative disease characterized by the infiltration of immune cells into the central nervous system and subsequent destruction of myelin [84,85]. Although the causes of MS remain unknown, the involvement of genetic and environmental factors in the development of MS has been widely suggested. In the last few years, degeneration of neurons has been linked to epigenetic modifications of neuronal and glial DNA [86,87]. Differentially methylated positions and regions have been found in immune cells and oligodendrocytes from MS patients compared to healthy controls [88,89]. Hypomethylation has been observed at the promoter region of peptidyl arginine deaminase 2 (PADI2), which catalyzes the citrullination of myelin basic protein (MBP). The result of the PADI2 hypomethylation is the inhibition of the MBP production, contributing to the loss of myelin stability in MS [90]. Interestingly, hypomethylation of PADI2 has also been observed in PBMC from MS patients [91]. Additionally, the reduction of methylation has been found in T cell IL-17A promoter, which results in an increased IL-17, and subsequently leads to the inflammation of the central nervous system [92,93].

Different histone modifications have been demonstrated to be involved in the transcriptional regulation in many of the cells engaged in the pathology of MS. It has been proven that there is a marked deacetylation in oligodendrocyte histones of MS patients, and the process is more frequent in chronic MS lesions than in the early stages of the disease [94]. Histone citrullination is another epigenetic modification implicated in the immunological process in MS. Citrullination of arginine 8 in histone H3 (H3Cit8) in PBMC prevents binding of the heterochromatin protein 1 (HP1) to neighboring H3K9me3 and leads to the inhibition of TNF-α and IL-8 production [95].

Epigenetic control in MS can also be achieved by microRNA-mediated gene silencing. The miRNAs, which are significantly upregulated in active MS lesions, are miR-326, miR-155 and miR-34a [96]. Overexpression of these miRNAs has been suggested to downregulate the expression of CD47, which diminishes the phagocytic activity of macrophages. miR-326 was found to promote the differentiation of naïve T cells into the T helper (Th)17 phenotype by targeting Ets-1, a negative regulator of Th17 differentiation [97,98]. Additionally, the expression of miR-326 is highly correlated with disease severity in both mice and humans suffering from autoimmune encephalomyelitis [97]. Noorbakhsh et al. demonstrated the upregulation of miR-155, miR-338 and miR-491 in brain samples of progressive MS patients and suggested their possible role in rendering white matter susceptible to inflammation-induced damage [99]. The expression of miR-155 was involved in autoimmune process by enhancing inflammatory T-cell development, therefore mice without miR-155 were resistant to development of experimental autoimmune encephalomyelitis (EAE), the most commonly used model for the human demyelinating disease [100]. Moreover, it was found that miR-96 overexpression was correlated with MS remission, whereas overexpression of miR-18b and miR-599 was associated with MS relapse [98].

### 4.4. Sarcoidosis

Sarcoidosis is a complex granulomatous disorder which is strongly related to immune response; however, its pathogenesis is still not completely understood. The immunological background of this disease involves the accumulation of activated macrophages and T-lymphocytes in affected organs, most commonly in the lung [101,102]. There is no information that epigenetic changes in monocytes could be associated with sarcoidosis, however both, occupational, environmental and infectious agents are involved in the development of this disorder [29].

In general, more proinflamatory cytokines (GM-CSF (granulocyte-macrophage colony stimulating factor), TNF-α, TGF-β-1 (transforming growth factor), IL-1β, IL-6, IL-17) and chemokines (chemokine C-X-C motif ligand (CXCL)9, CXCL10, CXCL11), reactive oxygen intermediate, chitotriosidase, angiotensin convertase enzyme and serum amyloid A have been observed among sarcoidosis patients. All of these molecules have been evaluated as biomarkers for the diagnosis, prognosis and response to treatment in sarcoidosis [29,103]. Interestingly, macrophages and circulating monocytes in patients with sarcoidosis express more CD16, TLR2 and TLR4 [29]. The activation of TLRs leads to the modulation of several adapter proteins such as IRAK (interleukin-1 receptor associated kinase) and Rip2 (receptor interacting protein 2). These kinases are pivotal for signaling pathways, including NF-κB and MAPKs (mitogen-activated protein kinases), which trigger cytokines production [29,102]. Beside macrophages T helper lymphocytes are present at sites of granulomatous inflammation in sarcoidosis. The activation of T CD4+ cells leads to the overexpression of IL-2R (IL-2 receptor) on the cell surface with the enzymatic cleavage of these molecules and release of sIL-2R (soluble IL-2 receptor) form [103]. Several studies have observed increased levels of sIL-2R in patients with sarcoidosis [104,105]. Genetic sarcoidosis risk factors have also been evaluated. Analysis of Fisher et al. revealed that granulomatous processes in sarcoidosis may be influenced by genes involved in the antigen presentation process (HLA alleles), immune cell activation (TNF, BTNL2 and IL23R) as well as apoptosis (ANXA11, XAF1) [106]. In the last decades the composition of the pulmonary microbiota in sarcoidosis has been discussed [107,108]. It is clear that pulmonary microbial community may be involved in antigen-driven T CD4+ lymphocyte activation followed by macrophage migration and granuloma formation. The 16sRNA gene analysis has revealed that pulmonary microbiota in sarcoidosis patients is composed of Firmicutes, Proteobacteria, Acinetobacter, Bacteroidetes, Fusobacteriales, and Spirochaetales. However, this composition does not differ from lung microbiota in patients with other interstitial lung diseases [107].

## 5. Neurodegenerative Disorders

The immune and nervous systems are a complex network of immune cells, including microglia (brain macrophages which are responsible for the elimination of microbes and production of proinflammatory cytokines), neutrophils, lymphocytes, neurons, astrocytes and oligodendrocytes [109,110]. In a healthy brain microglia provide synaptic plasticity through engulfing synaptic elements, including apoptotic cells, a process required for accurate brain development [111,112,113]. Microglia are able to modulate a wide spectrum of cellular responses and contribute to homeostasis as well as aging and neurodegenerative processes [114,115]. These cells retain a long-term memory of infectious and noninfectious agents, including stress [116]. Interestingly, in children exposed during prenatal life to a high level of the stress hormone cortisol persistent changes in the innate immunity have been observed, including overproduction of proinflammatory cytokines with further development of mental illness (behavioral and mental pattern) as well as psychological impairment. The susceptibility of the fetus to those factors may be due to the rapid changes in the nervous system [116,117]. The process of trained immunity, which takes place in the brain, is defined as microglial priming. Exposure to an initial stimulus (noxious or inflammatory factor as well as systemic illness or infection) induces the long-lasting memory of microglia. The secondary challenge of these cells causes an exaggerated inflammatory response, resulting in neuroinflammation and enhanced production of neurotoxic molecules [116,118,119]. The initial and secondary stimuli may be temporally separated. Many studies indicate that the inflammatory challenge in utero can be associated with impaired microglial reactivity in later life among offspring [120,121,122,123]. Püntener et al. revealed that repeated doses of LPS or a single injection of live *Salmonella typhimurium* SL3261 to mice intensified the IFN-γ, IL-1β and IL-12 production in the serum, spleen and brain up to three weeks post-infection [124]. Moreover, they observed the up-regulation of ICAM-1 (intracellular adhesion molecule-1), VCAM-1, major histocompatibility complex (MHC)I, and MHCII on the cerebral vasculature and the overexpresion of CD11b and CD68 on microglia. The consequence of the augmented level of these receptors on cerebral endothelium still remains unknown, however it can induce T cell tolerance, which contributes to the regulation of immune activation in the central nervous system [116,124]. Interestingly, elevated levels of proinflammatory cytokies, which are produced by microgila and other immune cells, can cause changes in behavior such as social withdrawal, loss of appetite, lethargy, pessimism, irritability and depression [125,126]. It is known that neurodevelopmental disorders can be associated with microglial dysfunction. The role of these cells has been studied in autism spectrum disorder (ASD), Alzheimer’s disease, accelerated aging, myotrophic lateral sclerosis, Parkinson’s disease and depression [114,115,118].

### 5.1. Alzheimer’s Disease

Alzheimer’s disease (AD) is a common form of dementia causing memory loss and impaired cognitive abilities as a result of extracellular accumulation of amyloid β (Aβ) and intracellular fibrillar Tau aggregation [114,127]. Recent large-scale genome-wide association studies (GWAS) have proved that the risk of developing late-onset AD is significantly associated with rare variants of innate immune receptors expressed on the microglia surface, such as CD33 (cluster of differentiation 33) and the triggering receptor expressed on myeloid cells 2 (TREM2). TREM2 is a transmembrane glycoprotein that is necessary for microglial survival, inflammatory response, and phagocytosis. TREM2 binds phospholipids during Aβ accumulation as a result of neuronal cell apoptosis and myelin damage [127,128]. Trem2-/- mice show invasiveness of fibrillary amyloid followed by the weakening of microglial barriers around Aβ [114]. CD33 is a transmembrane receptor of the innate immune system that is highly expressed on microglia and binds sialic acid, which leads to the activation of protein phosphatases SHP1 and SHP2. These phosphatases inhibit downstream signaling pathways resulting in hampering of microglial function, particularly phagocytosis of Aβ [127,129]. Interestingly, LPS-activated microglia eliminate viable neurons and synapses and promote the development of neurotoxic astrocytes A1 by releasing TNF-α, IL-1α and complement component C1q. All of these molecules activate A1 cells, which phagocyte healthy neurons and oligodenrdocytes [118,130]. Neutralizing antibodies to Il-1α, TNF-α, and C1q together inhibit the harmful activity of astrocytes. A similar effect was observed in knock-out mice (Il-1α−/−TNFα−/−C1q−/−) that failed to generate A1 cells [131]. These data provide evidence that astrocytes A1 are involved in human neuroinflammatory and neurodegenerative diseases.

The role of epigenetic factors, including DNA methylation, histone modifications and non-coding RNAs, in the development of AD has been widely studied. It has been found that demethylation of cytosines (from -207 to -182) in the promoter region of amyloid precursor protein (APP) gene may result in Aβ aggregation in the aged brain [132]. The study of Zhang et al. revealed that methylation of the microtubule-associated protein tau (MAPT) gene, especially in dinucleotide cytosine/guanine sites, triggers the function of microtubules and axonal transport [133]. These mutations lead to the formation of abnormal hyperphosphorylated tau protein, which participates in the formation of neurofibrillary tangles (NFTs) and induces abnormal earlier maturation and increased excitableness of neurons [134,135]. The biological activity of tau protein is also regulated by HDACs, which repress gene expression by condensing the structure of chromatin [132]. SIRT1, a member of the sirtuin family of nicotinamide adenine dinucleotide (NAD)-dependent HDACs, catalyzes the deacetylation of lysine 28 in the microtubule-stabilizing protein tau, which inhibits its function and promotes its aggregation [136,137]. Julien at al. observed that a significant reduction of SIRT1 level in the brains of AD patients was associated with the accumulation of Aβ and tau protein [138].

### 5.2. Parkinson’s Disease

Parkinson’s disease (PD) is an adult onset neurodegenerative disease characterized by selective degeneration of neurons, including loss of dopaminergic neurons and the presence of Lewy bodies containing α-synuclein, which results in motor and nonmotor symptoms [139,140,141]. α-synuclein plays a leading role in the initiation and progression of neurodegeneration as a result of neurotoxicity induction by various pathways, such as inflammation, oxidative stress as well as autophagy abnormalities [141,142]. TLR1 and TLR2 on microglia recognize α-synuclein followed by the activation of phagocytic TAM receptor tyrosine kinases: Axl and Mer [143]. The functional roles of these receptors include regulating the innate immune response with phagocytosis of distressed spinal motor neurons and controlling the target genes involved in the homeostatic regulation of TLR-mediated signal transduction pathways [114,143,144].

Differences in DNA methylation, histone modifications and miRNA expression are also relevant in the dysregulation of the expression of some genes, which are important in the pathogenesis of PD. The expression of α-synuclein is regulated by DNA methylation. The addition of a methyl group to CpG islands in the first intron (5′-regulatory regions) of the α-synuclein gene results in transcriptional repression. Jowaed et al. and Matsumoto et al. observed that demethylation of the α-synuclein gene affects the pathogenesis of Parkinson’s disease [145,146]. Moreover, the Dntm1 (DNA methyltransferase 1) level, one of the key regulator enzymes that inhibit DNA methylation, has also been found to be lower in human postmortem cortical brain samples from PD [147]. Methylation mechanisms have also been analyzed in mRNA. The post-transcriptional modifications relate to N6-methyladenosine and 5-methylcytosine. The consequence of the mutations in FTO (Fat mass and obesity-associated) gen, that encodes nucleic acid demethylase, is the impairment of dopaminergic neuron activity via dopamine receptor type 2 (D2R) and dopamine receptor type 3 (D3R), which has been observed in mouse brain model and among patients with Parkinson’s disease [148,149]. Chromatin remodeling, including methylation and acetylation of histones, is associated with transcriptionally active genes. α-synuclein can bind to H3, which prevents histone acetylation, inhibits gene expression, leads to α-synuclein fibrillation and toxicity, and in consequence results in cell death [149,150]. Many environmental neurotoxins, such as dieldrin or paraquat, which trigger the pathogenesis of PD, are related to hyperacetylation of H3 or H4 in dopaminergic neurons [151]. Choi et al. revealed that miR-7 represses α-synuclein expression by targeting the 3′-untranslated region of its mRNA as well as by facilitating the degradation of α- synuclein protein/aggregates by promoting autophagy, which was evaluated in the human neural progenitor cell line ReNcell VM [152,153]. Similar effect was observed by Doxakis at el., who concluded that miR-7 and miR-153 play a role in modulating α-synuclein protein levels in the nervous system, which can be considered as a potential therapeutic strategy for PD [154].

### 5.3. Autism Spectrum Disorder

Microglia dysfunction may also contribute to the development of autism spectrum disorder (ASD). It is a neuropsychiatric condition characterized by impaired social communication, including restricted interests, stereotyped and repetitive behavior or cognitive disabilities [115,155,156,157]. The causes of ASD still remain unknown, but many studies indicate a high genetic contribution. Molecular analysis revealed over one hundred ASD chromosomal rearrangements that are present in as many as 20% of affected individuals [155,158]. The genes associated with ASD are involved in multiple cellular functions, including chromatin remodeling, metabolism, mRNA translation, and synaptic function as well as neuronal/synaptic homeostasis [158,159]. Furthermore, several studies determined that pro-inflammatory skew, including autoimmune background, can lead to this disease [115,160]. Cytokines may affect behavior through effects on neurotransmitter function, neuroendocrine activity and neurogenesis. These processes are associated with the abnormalities in the balance between excitatory (glutamate-mediated), and inhibitory (GABA-mediated, gamma-aminobutyric acid) neurotransmission, which can result in the pathological process of excitotoxicity [161,162]. It is known that maternal immune activation to an invading pathogen during the first trimester of pregnancy increases the risk of ASD in children. One of the hypotheses that attempts to explain this reaction is based on the evidence that maternal infection leads to the release of pro-inflammatory cytokines and activation of Th17 cells, which affects the immune status and genetic predisposition of the fetus. Analysis of the cytokine profile at birth revealed elevated IL-1β and IL-4 levels, which can be considered as a prenatal immune challenge, followed by the development of ASD symptoms later in childhood [163,164].

## 6. Conclusions

The term “trained innate immunity” describes a kind of immunological memory which is completely distinct from the classical one, represented by a highly specific and potent response of lymphocytes. After primary stimulation, innate immune cells develop a trained immunity phenotype, which allows them to respond more effectively to subsequent restimulation with related or unrelated stimuli. Unlike in the case of classical adaptive response, where the gene rearrangement plays a pivotal role, trained immunity depends on the epigenetic reprogramming with which the changes in cellular metabolism are often closely connected. The phenomenon of innate immune memory possesses the potential for helping to design some activators of innate immune response or new generation of vaccines. However, there is some evidence indicating the contribution of epigenetic reprogramming to the occurrence of different detrimental processes, including those connected with the development of atherosclerosis, diabetes mellitus, autoimmune disorders or neurodegenerative diseases. The epigenetic mechanisms accompanying the induction and progression of many pathological processes have been only partially identified, and their participation in the development and maintaining of this process should be considered circumstantial or presumptive.

## Abbreviations

ABCA1	cholesterol efflux regulatory protein
ABCG1	intracellular cholesterol transporting protein
BCG	*M. bovis* BCG (Bacillus Calmette-Guérin)
GM-CSF	granulocyte-monocyte colony stimulating factor
CXCL	chemokine (C-X-C) ligand
DNMT	DNA methyltransferase
HAT	acetyltransferase
HDAC	histone deacetylase
HDM	histone demethylase
HLA	human leukocyte antigen
HMT	histone methyltransferase
ICAM-1	Intracellular adhesion molecule 1
IFN	interferon
IL	interleukin
IRAK	interleukin-1 receptor associated kinase
LPS	lipopolysaccharide
MBP	myelin basic protein
MCP-1	monocyte chemoattractant protein-1
MHC	major histocompatibility complex
MMP	metalloproteinase
NK	Natural killer cell
oxLDL	oxidized low-density lipoprotein
PADI2	peptidyl arginine deaminase 2
Pam3Cys	tripalmitoyl-S-glyceryl cysteine
PPARG1	pepxisome proliferator-activated receptor gamma
ROS	reactive oxygen species
SUMO	small ubiquitin-like modifier
TGF-beta	transforming growth factor
Th	T helper cell
TLR	Toll-like receptor
TNF	tumor necrosis factor
TSA	trichostatin A
VCAM-1	vascular cell adhesion molecule 1
